# Born Too Soon: Accelerating change to 2030 and beyond

**DOI:** 10.1186/s12978-025-02035-9

**Published:** 2025-06-23

**Authors:** Joy E. Lawn, Rajat Khosla, Amy Reid, Etienne V. Langlois, Mary Kinney, Gagan Gupta, Doris Mollel, Bo Jacobsson, Maria El Bizri, Anna Gruending, Harriet Ruysen, Kelly Thompson, Per Ashorn, Lori McDougall, Helga Fogstad, Fouzia Shafique, Anshu Banerjee

**Affiliations:** 1 Maternal, Adolescence, Reproductive, & Child Health (MARCH), London School of Hygiene & Topical Medicine, London, UK; 2 Partnership for Maternal, Newborn and Child Health (PMNCH), World Health Organization (WHO), Geneva, Switzerland; 3 School of Public Health, University of the Western Cape, Bellville, South Africa, and Global Surgery, University of Cape Town Faculty of Health Sciences, Observatory, Western Cape, South Africa; 4 UNICEF, New York, USA; 5 Doris Mollel Foundation, Dar Es Salaam, United Republic of Tanzania; 6 Department of Obstetrics and Gynecology, University of Gothenburg, Gothenburg, Sweden, Department of Obstetrics and Gynecology, Western Health Care Region, Sahlgrenska University Hospital, Gothenburg, Sweden; 7 Department of Maternal, Newborn, Child and Adolescent Health and Ageing, World Health Organization, Geneva, Switzerland

**Keywords:** Preterm birth, Maternal, Neonatal, Global health, Financing, Investment, Innovation

## Abstract

**Progress needed:**

Preterm birth rates have “flatlined” for a decade with major loss of human capital, hindering progress for many Sustainable Development Goals. Progress on the reduction of maternal, newborn and child mortality needs to accelerate by between 3 and 11-fold to reach national and global targets by 2030.

**Priorities:**

Actions are required on two tracks: (1) prevention of preterm birth, including better management for women in preterm labour, and (2) provision of high-quality care to vulnerable newborns, including those born into fragile and conflict-affected settings. Together these tracks have potential for high impact in terms of millions of lives saved, and socioeconomic returns on investment. We can and must do more to provide quality and respectful reproductive, antenatal and birth care for all adolescent girls and women, everywhere, and close unacceptable survival gaps for small and sick newborns. New focus is essential on threats beyond the health sector, notably conflict and the climate crisis.

**Pivots:**

The cost of inaction is too high in every country. Four pivots are central to accelerating action: invest, implement, integrate, and innovate. More specifically these pivots include investments in systems including more skilled human resources; implementation of high-impact interventions with data used for quality improvement and accountability; innovations including new health technologies and also systems and social innovations; plus, integration with levels of the health sector and across sectors and the life-course, with families at the centre. Everyone has a role to play. Increasing speed now, and sustaining progress, requires multi-level leadership including from grassroots movements led by parents and affected people through to heads of state. Some countries provide examples of such change: The United States of America in data identified inequalities by state and ethnicity for preterm birth. Importantly noting drops in donor aid, India has made ambitious investment in the health sector and beyond, and United Republic of Tanzania in multi-level leadership. Changing gears requires the ambition and energy witnessed a generation ago for HIV/AIDS. We have the ability now to ensure that every baby born too soon – and their mothers – can survive and thrive. Our next generation depends on us acting now for more healthy starts and hopeful futures.

## Key findings

### 1. Investments increased

More strategic investments are urgently needed to address critical gaps in health systems that affect outcomes for small and vulnerable newborns. The proportion of current donor investments in reproductive, maternal, newborn, and child health services and interventions remain insufficient considering the substantial burden of preterm birth, despite clear evidence showing significant returns in human capital and national economic growth. Only 22% of 106 high-burden countries reported having a budget line for small and sick newborn care.

### 2. Implementation accelerated

Families and communities must be meaningfully engaged at all system levels—from health facilities to the design of national and subnational policies and programs, as well as global platforms focused on maternal and newborn health. Resources and coordinated action are urgently required to implement evidence-based solutions and scale up proven interventions through collaboration among governments, humanitarian organisations, and local communities, also considering the role of the private sector.

### 3. Integration enabled within health and other sectors

Beyond health systems, integration must also extend to five key intersectoral domains to prioritise maternal and newborn health: equity and rights, environment, economy, education, and emergencies.

### 4. Innovations developed and implemented

Focus is needed on innovations, both products and programmatic, that improve maternal and newborn health outcomes. Given the potential to reduce burden, smarter investments are needed, including those that facilitate locally led research and implementation.

## Purpose

This is the final paper of a series of seven which were developed from the report “*Born Too Soon: A decade of action on preterm birth”* [1]. The report highlighted stagnating progress for maternal and newborn health and stillbirths and underlined the lack of progress for preterm birth as foundational. Content in that report and these papers included new data, literature reviews and case studies, synthesized into three themes in each paper: (1) progress in the last decade; (2) programmatic priorities based on the evidence; and (3) pivots to accelerate progress in the decade ahead. Preterm birth refers to babies born before 37 completed weeks of gestation. The first paper outlines more details on the definitions and terminology [2].

This paper defines the two tracks of action needed to reduce the burden of preterm birth: first, the prevention of preterm birth, and secondly, care for an estimated 13.4 million babies born too soon per year [3]. Given the limited progress in preventing preterm births, there is an urgent need for innovation [4]. For babies born preterm, significant impact can be achieved by closing the survival gap between low- and high-income settings [5]. Changing gear and accelerating quality care would enable more countries to meet national targets by 2030, while also transforming human capital. The paper highlights the pivots required to drive this accelerated progress.

## Progress needs to accelerate

Preterm birth is a global issue with high rates across all regions and has contributed to the slowing progress in maternal, newborn, and child health (MNCH) over the past decade, at a time when accelerated advancements were critically needed.

**Fig. 1:**
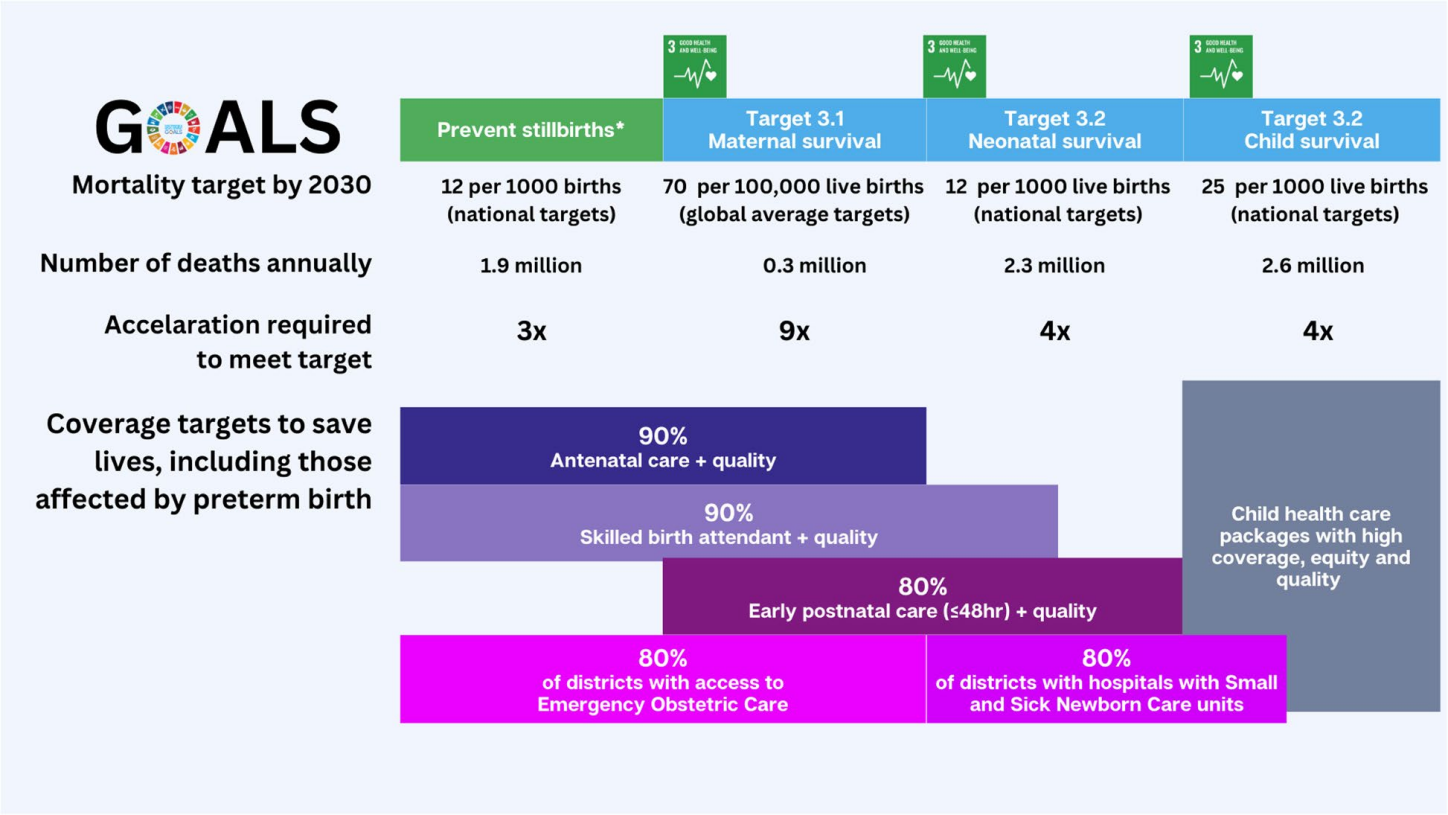
Status for maternal, newborn and child deaths plus stillbirths compared to 2030 mortality targets, and coverage of care targets to accelerate progress. *stillbirth target is in ENAP [10] and Global strategy [11] but not in SDGs. Adapted from WHA77 Resolution on MNCH and the Road to reducing maternal, newborn and child mortality [12] and coverage targets set by ENAP/EPMM, now called EWENE (Every Woman, Every Newborn, Everywhere) [7]. EWENE coverage indicators for antenatal care, skilled birth attendant and postnatal care have linked quality metrics.

During the Millennium Development Goal era, concerted investments and inclusive partnerships at all levels led to reductions in maternal and child mortality. However, major inequalities remained, and progress has slowed, particularly for deaths occurring closer to the time of birth. Each year, over 7 million preventable deaths continue to occur: last trimester stillbirths (1.9 million), neonates (first 28 days, 2.3 million), children from 1 month to 5 years (2.6 million) and of women due to maternal conditions (0.3 million) [6, 7]. Africa has 16% of the world’s population but more than half of the burden of these deaths [8]. Achieving the Sustainable Development Goals (SDG) targets by 2030 requires a transformative acceleration in progress—approximately a three- to four-fold increase for stillbirth, neonatal, and child mortality rates, and a nine-fold increase to meet the global average target for the maternal mortality ratio (Fig. 1) [9].

Rates of preterm birth are high, affecting one in ten births, and progress has “flatlined” with no measurable change in the last decade in 52 countries, while preterm rates have risen in 13 countries [3, 13]. Among the few countries showing progress, the reduction rate is only 0.5% per year [3]. While lower-income countries bear a disproportionate impact, middle- and high-income countries are also affected [3]. The lack of progress in addressing preterm birth and its causes is hindering progress for maternal, newborn, and child health. Direct complications of preterm birth are the leading cause of under-five child deaths, with almost one million deaths per year, and rank high in the global burden of disease across all conditions, resulting in substantial losses in human capital [14].

Preterm birth has lifelong effects on survivors, impacting over 13.4 million newborns annually, [3] along with significant consequences for their families and countries [15]. Babies born before 37 weeks face an elevated risk of impairment with an estimated 8% of survivors having neurodevelopmental impairment and many more having reduced vision due to Retinopathy of prematurity [6]. Long-term outcomes of preterm birth include reduced cognitive performance, heightened risks of depression and behavioural conditions during adolescence, more susceptibility to developing chronic diseases later in life, such as hypertension, and risks of decreased employment opportunities and quality of life in adulthood [16].

Families of preterm babies often face significant mental health challenges, including post-traumatic stress disorder [17]. The broader community also feels the effects, such as lost earnings and missed employment opportunities associated with caring for a preterm baby, which are particularly burdensome in contexts with high out-of-pocket healthcare costs. These challenges affect families in multiple ways, including the education, health, and care of other children. For example, in the United States of America, medical debt is a significant driver of bankruptcy overall, with pregnancy related costs being a major contributor [18–20]. Moreover, families faced with preterm infants can expect 10 times the associated medical costs of a full term pregnancy [21, 22,23].

Health systems face a significant burden from high rates of preterm birth and associated outcomes, such as stillbirth. Extremely preterm babies often require specialised care, including prolonged hospital stays. Caring for preterm babies and their families also impacts healthcare professionals, with vulnerable newborns sometimes dying within minutes—making such care a critical marker of a responsive and resilient health system.

At national level, vulnerable newborns represent an enormous loss of human capital and potential for economic growth [15]. Failing to provide appropriate care has far-reaching consequences, reverberating not only throughout the life course but also across generations, resulting in staggering human and economic costs.

 Earlier estimates showed that Investing in high coverage and quality maternal and newborn care in 75 high-burden countries between 2015-2025—at a cost of just US$1.15 per person per year—could have prevented an estimated 71% of neonatal deaths, along with significant reductions in stillbirths and maternal mortality [24]. Ensuring the continuum of care extends from communities to hospitals is critical, as adequately supported and resourced community-based platforms can avert an estimated 49% of newborn deaths [25].

To accelerate progress, the World Health Organization (WHO), UNICEF and the United National Population Fund (UNFPA) launched joint “Every Woman, Every Newborn Everywhere” (EWENE) coverage targets for maternal and newborn healthcare packages worldwide. These four targets include 90% coverage for antenatal care, 90% for childbirth care, 80% for postnatal care, and ensuring that 80% of districts or their equivalent have functional small and sick newborn care units as well as closely linked emergency obstetric care (Fig. 1). Achieving high coverage and quality of these four packages as part of primary health care, including district hospitals, is fundamental to ensuring faster progress for MNCH, including addressing preterm birth [7]. EWENE has defined quality-of-care metrics to add to these coverage indicators. Local action is essential for driving national and global change.

## Priorities for impact

**Fig. 2:**
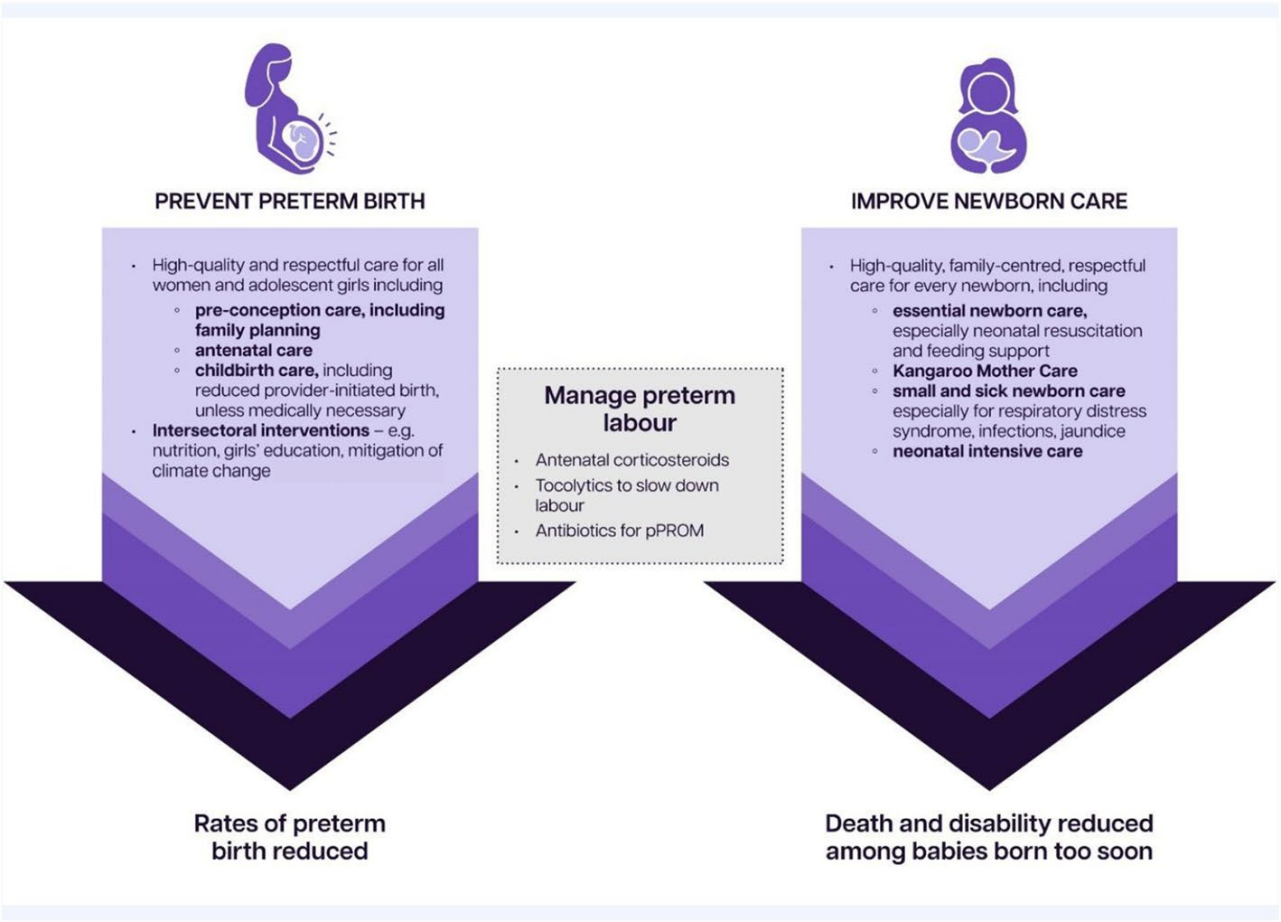
Twin tracks to reduce the burden of preterm birth. Adapted from Born Too Soon Report 2023 [1]. Abbreviations: pPROM: preterm prelabor rupture of membranes

To reduce the burden of preterm birth and unlock human capital, two priority tracks for action have been identified: (1) preventing preterm birth and (2) improving care for those affected (Fig. 2). As the figure shows, there is also an important link between the two tracks for care at the time of preterm birth. Both tracks involve placing women, newborns, and their families at the heart, ensuring their rights to respectful, high-quality care are upheld, including engaging them meaningfully in decision-making and care provision.

### Track 1: Prevent preterm birth

Accelerating progress to reduce preterm birth requires targeted action on known risk factors and strategic investments in research to better understand preterm births with no identifiable causes. Protecting sexual and reproductive health and rights (SRHR) is essential, along with addressing gaps in both the coverage and quality of care across the continuum. This includes more investments in family planning plus obstetric care and midwifery [4, 26]. Explicit efforts are needed to reach adolescents, providing them with sexual and reproductive health services and supporting adolescent mothers, who face higher risks themselves during pregnancy, birth and beyond, and whose babies are also more likely to be preterm or stillborn [27]. In addition, addressing child marriage, is crucial.

WHO recommends antenatal care for all pregnant women to facilitate access to health services and initiate positive relationships with healthcare providers, as well as planning for transport if needed. High quality antenatal care is an opportunity to identify and address risk factors for preterm labour, such as maternal infections including HIV and malaria or obstetric complications like pre-eclampsia [28, 29]. Undernutrition, including anaemia, zinc and calcium deficiencies and low body mass index (BMI), is also a key contributor to preterm birth [30, 31].

Changes beyond the health sector, such as education, can positively impact girls’ health since those who remain in school are more likely to delay sexual debut and less likely to experience early marriage. Air pollution levels are associated with increased preterm birth, and evidence shows a rise in preterm birth by 4% for every 1 °C in temperature and by 26% during heatwaves [32, 33]. Taking measurable steps towards improving air quality and addressing the impacts of climate change can provide direct health benefits to women and their babies [34]. This is also explored further in Paper 6 of this supplement [34]. In addition, information about what pollutants and levels of heat increase risk should be made available to families so they can mitigate exposure where possible.

In between the two tracks of prevention and care (Fig. 2), the important moment of an anticipated preterm birth gives opportunities to seize to improve outcomes for both the woman and her baby, including the appropriate use of antenatal corticosteroids and tocolytics, as well as increased fetal monitoring especially for the most vulnerable such as multiple pregnancy [4].

### Track 2: Provide high-quality care for small and sick newborns

The survival gap for preterm babies globally mirrors the HIV survival gap seen two decades ago, which was dramatically narrowed through concerted efforts to ensure access to anti-retrovirals [35]. Among preterm newborns who receive high-quality care, even those born at just 28 weeks of gestation (three months early), more than 95% survive without severe disability [5]. However, of those born in settings without such care, fewer than 5% survive [36].

Yet there is evidence and examples of impactful interventions and improvement. For example, the 10 countries making the most rapid progress in newborn survival have dramatically reduced their neonatal mortality rates in the last decade by investing in SSNC [37]. Reaching the SDG goal of a neonatal mortality target of < 12 per 1000 livebirths globally will depend on high coverage of SSNC, including for preterm babies needing respiratory support. Impact requires the right space, the right people (e.g. more neonatal nurses), the right devices and drugs including supply chain, and the right data to monitor and improve care. Scale up learning has been codified into 10 core components of systems change, also including strengthening referral systems as detailed in paper 5 of this series [5].

We also need evidence-based innovations that empower and engage women and their families, such as Kangaroo Mother Care (KMC) and family-centred care. Local action is essential to bringing improved newborn care units to every district of every country.

## Pivots for collective action

To reduce the burden of preterm birth and optimise high-quality maternal and newborn care in the next decade, this paper outlines four key actions; invest, implement, integrate, and innovate.

### Action 1: invest

Increasing investment is essential to meeting the SDG targets for maternal and newborn survival and the Global Strategy target for stillbirth prevention [38]. Allocating resources to maternal and newborn health is not just an expenditure—it is a highly effective investment with substantial returns. While the financial commitment needed to advance these two tracks is significant, the returns are huge.

Smarter investments are crucial from governments, leveraging both government and domestic resources, as well as donor contributions. While national investments are often larger than donor inputs, their lack of transparency makes tracking difficult, weakening accountability mechanisms. The official development assistance (ODA) funding for reproductive, maternal, newborn and child health (RMNCH) interventions were already decreasing prior to the COVID-19 pandemic and further decreased by 14%, from US$ 6.2 billion in 2019 to US$ 5.3 billion in 2021 [39]. Funding which mentioned the word *newborn*, mostly as included in the phrase *MNCH,* represented just 10% of reproductive, maternal, newborn and child health allocations overall [40]. Less than 1% of RMNCH funding was allocated to specific interventions targeting newborn health, such as KMC or breastfeeding, despite 2.3 million neonatal deaths worldwide each year [41] accounting for about one-third of MNCH-related deaths. Stillbirths programs received an even smaller fraction of funding, at a mere 0.0003% of the total, despite 1.9 million deaths worldwide annually [40].

The response to COVID-19 has highlighted the importance of investing in research and robust health data systems as a foundation for effective countermeasures. These investments, made for COVID-19 and other pandemics, can be leveraged for example for infection prevention, laboratory strengthening and oxygen systems, all of which are critical for addressing the burden of preterm birth [34, 37, 42].

It is essential to integrate both maternal and newborn care services into Universal Health Coverage (UHC) policies ensuring access to high-impact maternal and newborn health interventions alongside financial and social protection measures. Catastrophic health expenditures can push families into poverty, discourage them from seeking necessary health services, and, in some cases, lead to mothers and babies being detained in facilities until payments are made, being refused care or not seeking care because of insurmountable financial barriers [37]. While recent data from 106 high-burden countries, found 71% were exempt from user fees, 31 countries still offered no exemption leaving vulnerable women and their families at risk of significant financial burden through pregnancy and beyond [7].

Investments yield the highest returns when focused on high-quality care for women before, during, and after birth, as well as in newborn care. This forms the backbone of a robust primary health care system, including ensuring that every district hospital can provide emergency obstetric care and small and sick newborn care. In settings where only a small, inadequate room is available, respectful and high-quality care cannot be delivered effectively. Building new, well-designed facilities that can last for decades (economic amortisement usually over 20 years) is generally assumed by economists to be more cost-efficient than multiple major renovations every few years [43, 44]. Such facility development should be designed to support the shift towards family-centred care, enabling parental partnership in caring for a preterm newborn [45]. Health infrastructure development that is both climate-resilient and low-emission are additional considerations for sustainability [45]. While the initial costs may be higher, the long-term benefits are substantial, making these investments not only cost-effective but also essential for sustaining progress in reducing mortality as simpler interventions are scaled up and more advanced care becomes needed [36, 46].

Increased and smarter investments are urgently needed. Transformational system changes are required, including increasing the number of midwives and nurses, strengthening data systems, and ensuring sustainable biomedical device systems—rather than simply placing donated equipment that often ends up unused in "equipment graveyards." High-quality care is unattainable without increased financial investment. Crucially, these investments yield significant returns, not only in human capital across the life course but also in driving national economic growth. For instance, through such strategic investments, China has achieved the fastest annual reduction rate in neonatal mortality globally, at 8.8% per year [36].

**Fig. 3:**
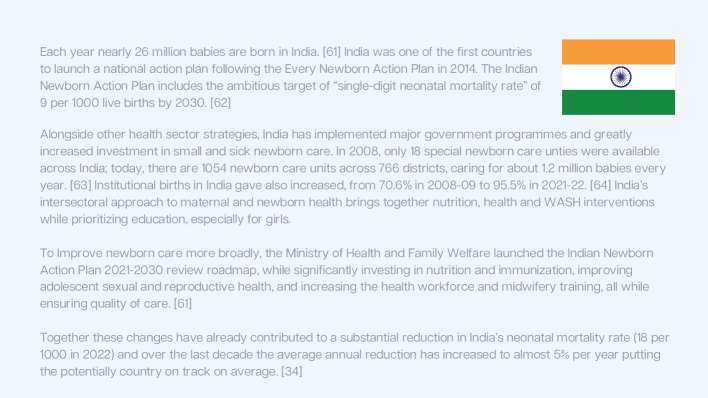
Country snapshot: Government-led ambitious investment and intersectoral change in India. References [41, 48–51]

India has set a national target for neonates to achieve a neonatal mortality rate of 9 per 1,000 live births, surpassing the SDG 3.2 target of 12. This ambitious goal has been matched with significant nationwide investments, including scaling up special care neonatal units from approximately 10 to nearly 1,000 over the past 15 years. These efforts encompass infrastructure development with standardized floor plans, substantial investments in human resources, and the implementation of a unified national dataset (Fig. 3). Additionally, India has prioritised maternal care and leveraged intersectoral platforms addressing education, gender equality, and nutrition. Over the last five years, the pace of neonatal mortality reduction has accelerated, with the average annual rate of reduction doubling [47].

For 74 high-burden countries, every US$ 1 spent on key interventions for RMNCH is estimated to lead to a return of between US$ 9 to US$ 20 in economic and social benefits as a result of lower morbidity and mortality between 2015- 2035 [52]. Across South Asia, scaling up a package of interventions including small and vulnerable newborn care, has been calculated to return US$ 2–17 for every US$ 1 invested [53]. In the United Republic of Tanzania, an investment case for SSNC national scale up showed the potential to get on track for SDG3.2 and a comparable potential return on investment of between US$ 7 and US$ 9 for every US$ 1 invested [6]. Globally, breastfeeding is one of the most cost-effective interventions, returning US$ 35 for each US$ 1 invested in its promotion and protection [54].

Despite the potential return on investment, financial commitments have remained insufficient. Shiffman and others have described three elements that influence the prioritisation of an issue: the power of the actors involved, the strength of the narrative or ideas, and the broader contextual factors [55]. Women's and children's health issues have often been deprioritized, partly due to the limited influence of key advocates and the persistent stigma faced by women who experience stillbirths or neonatal deaths [56]. Global stakeholders must play a pivotal role in advocating for mothers, babies, and families while also driving the adoption of new and innovative solutions.

### Action 2: implement

Adopting the right policies is essential but not sufficient. While many countries have established targets and national policies, implementation remains a significant challenge. Local action is critical to driving national and global progress, as emphasised in the WHO/UNICEF report on progress toward maternal and newborn health coverage targets [7].

Achieving impact requires systemic change in every district of every country. Ensuring access to a high-impact package of SSNC WHO Level-2 care, including continuous positive airway pressure (CPAP), is a critical strategy. High-quality care is essential for achieving impact, alongside high coverage. EWENE targets underline the priority to scale up the core SSNC WHO level 2 package to every district, every woman and every newborn, maximising impact and equity. More complex, expensive interventions (e.g. surfactant) can be scaled up once equitable coverage of high-quality level 2 SSNC has been achieved, and mortality transition has progressed. Adding such interventions early for some could be less effective as the basics are not in place, and may exacerbate inequalities, for example if it is only provided in national referral hospitals. Availability of a core package of high-impact interventions for all women and babies is central to a driving change with equity.

**Fig. 4:**
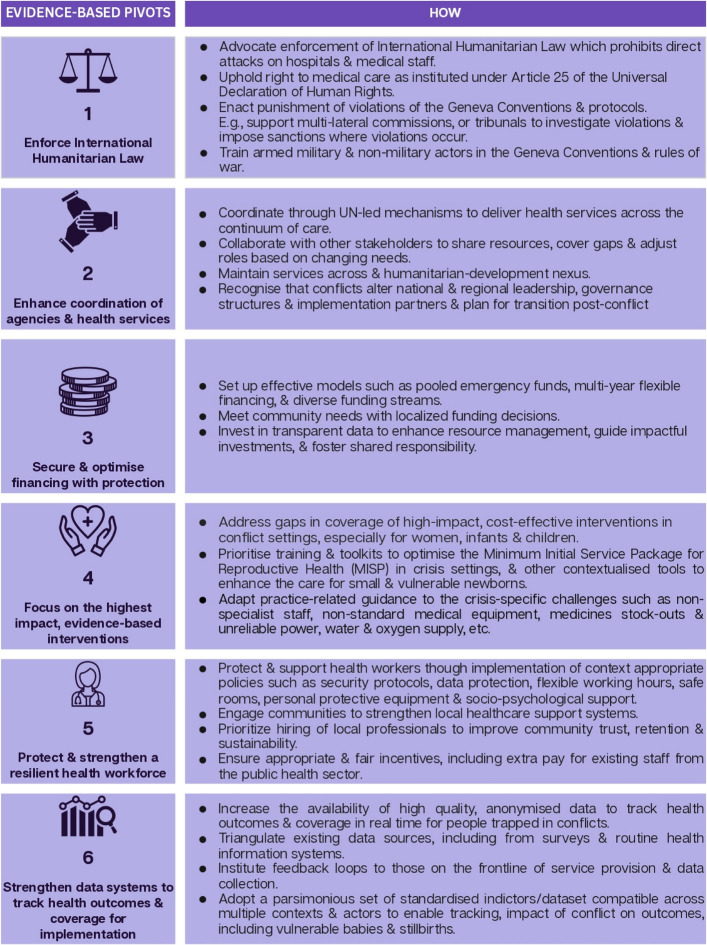
Implementation learning for actions in conflict settings to protect women, children, and vulnerable newborns. References: [59–66]

Conflict exacerbates vulnerabilities, deepening poverty, malnutrition, displacement, and trauma while dismantling essential support systems for marginalised populations, including women, children, and newborns [57, 58]. Preventable causes of mortality increase due to inadequate healthcare, poor nutrition, and the collapse of maternal and child health services. Urgent resources and coordinated action are needed to implement evidence-based solutions and scale up proven interventions that integrate health, nutrition, education, protection, and social support systems. This requires collaboration among governments, humanitarian organisations, and local communities. Figure 4 showcases evidence-based pivots to protect vulnerable women, newborns and children living in conflict settings.

Much has been achieved, but much remains to be done: not just listening to women and parents, but fully partnering with them at all levels of healthcare: from individual facilities to national and subnational policies and programmes, and on global platforms for maternal and newborn health more broadly [67].

### Action 3: integrate

The activities of health systems and other sectors have both direct and indirect impacts on preterm birth and the survival of preterm babies. Integration remains key to accelerating progress.

Preterm birth must be addressed within the continuum of care, ensuring that high-quality care supports a woman and her newborn across time—from pre-conception, at birth and continuing through childhood, adolescence, pregnancy, and beyond [68]. Integration across the life course has also additional value-for-money benefits, through packaged low-cost interventions [69]. Essential services that directly address preterm birth prevention and the care of vulnerable newborns must be prioritised within primary health care.

Beyond the health system, Paper 6 in this Born Too Soon series [34] identified five intersectoral domains impacting maternal and newborn health, especially for the most vulnerable: equity and rights; environment; economic; education; and emergencies. The climate crisis poses a major but underappreciated threat to pregnant women and newborns. Intersectoral interventions significantly contribute to maternal and newborn health outcomes. For instance, education for girls and women improves health-service utilisation, delays age of first pregnancy and marriage and reduces rates of preterm birth and maternal risk. Similarly, WASH (Water, Sanitation, and Hygiene), nutritional interventions, and social protection are critical, especially in addressing intersectional inequities [34].

**Fig. 5:**
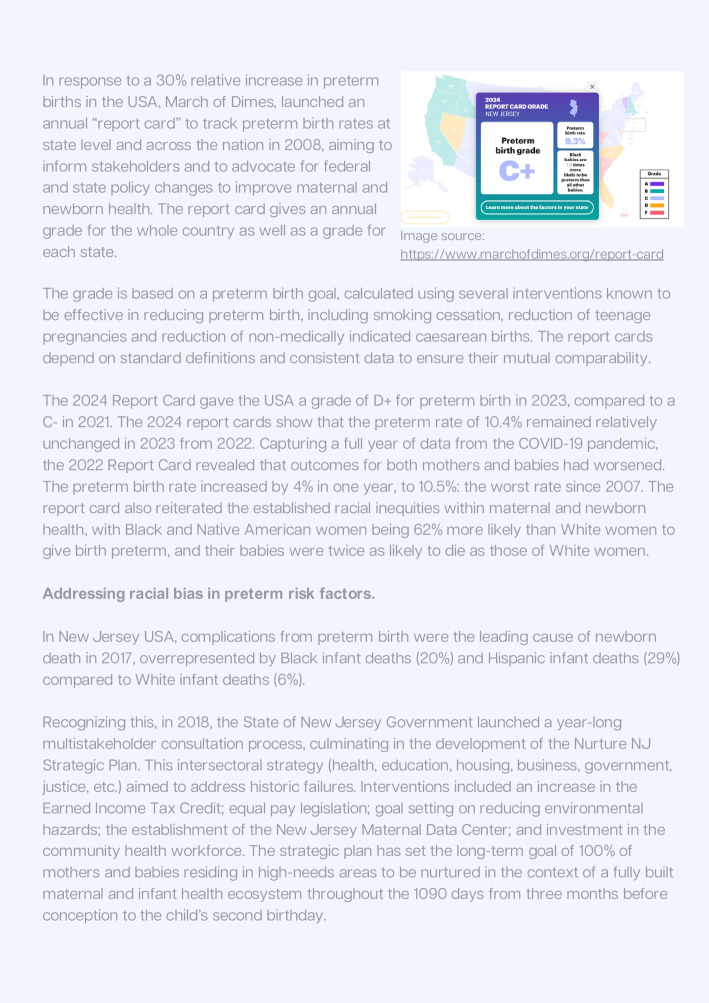
Country Snapshot: Assessing and addressing racial disparities in preterm birth risk factors in the United States of America (USA). References: [71–73]

Data are pivotal for driving accountability and action across programmes. Figure 5 illustrates the use of preterm birth and inequality report cards to address racial inequities in maternal and newborn health in the United States of America [70]. This approach underscores the importance of measuring and recording stillbirth and preterm birth in programmes and research to deepen our understanding of intersectoral risks and interventions that impact preterm birth and other adverse outcomes.

### Action 4: innovate

Technical innovations are critical to delivering high-quality maternal and newborn health care. However, innovation is not limited to technological products; it also includes programmatic systems change, social participation, digital/data shifts and innovative financing [74]. Locally led innovation is essential for closing implementation gaps and can be supported through multi-country learning networks and meaningful engagement with communities and parent groups.

**Fig. 6:**
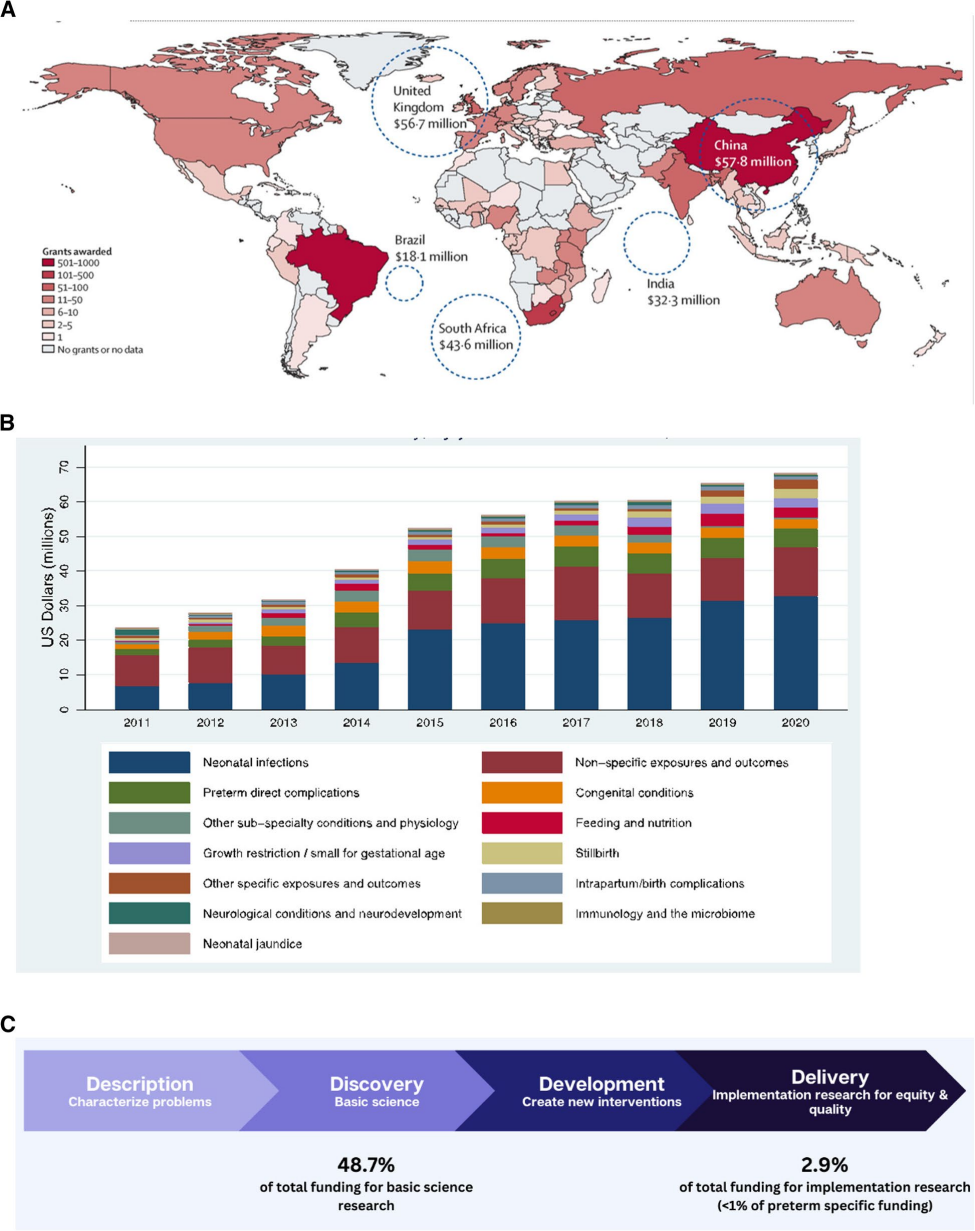
Global research funding with a focus on Low- and Middle-income countries (LMIC) for neonatal health and stillbirths, highlighting spending on preterm birth research funding. **A** Number of active grants received by at least one LMIC partner, 2011-2020 showing total amount, noting 32% co-recipient in a high-income country. Reference: [75]. **B** Research funding to LMIC trends from 2011-2020, and by focus of topic, showing increased funding over time and most focus on neonatal infections and preterm birth. Reference: [75]. **C** Research funding to LMIC partners, 2011-2020 total $486·7 million, according to research type split by the innovation pipeline. Reference: [75]

Smarter research funding is urgently needed, aligned with the burden of disease and potential for impact. Analyses of global research funding shows that US$ 577 million per year is directed towards neonatal outcomes; however, significant equity gaps remain (Fig. 6) [75]. While 98% of the burden of neonatal deaths and stillbirths occurs in low-and-middle income countries (LMICs), less than 7% of global funding is directed to these regions. About one third of this funding is funnelled through institutions in high-income countries [75]. Encouragingly, some middle-income countries, notably Brazil, China, and South Africa, are increasing their investments in preterm birth research in their own countries [75].

The research priorities also reveal disparities: while infections and preterm birth are the most researched topics, stillbirth research receives less than 3% of funding despite almost two million deaths per year [75]. Similarly, implementation research—which bridges the gap between evidence and practice and has the potential for immediate, high-impact response—receives less than 3% of funding, with the majority of investments being in basic science [75]. To achieve meaningful health outcomes rather than academic outputs alone, research funders must be held accountable for smarter investments. This includes prioritising locally led research in regions with the highest burden and focusing on evidence-based implementation strategies to maximise impact.

## Accelerating change: top- down and bottom-up for greater momentum

National actors must work with global partners to prioritise action, advocate and invest. Only through joint efforts can stakeholders ensure that every woman and adolescent girl receives high-quality, respectful care and that every baby, everywhere, “has a chance to be born at the right time, and the right size,” and to survive and thrive [76].

Achieving these goals requires pressure at all levels to develop subnational plans and decentralise resources. These efforts must flow not only from the top down (governments and donors) but also from the bottom up, through families, communities, health-care providers, and civil society. Additionally, engagement is needed from the middle, encompassing policymakers and those implementing maternal and newborn health services. By working together, stakeholders can amplify advocacy efforts and hold leaders accountable.

Collaborative action has the potential to shift social norms. Leaders, parliamentarians, policymakers, and even heads of state must act decisively on behalf of their most vulnerable citizens to drive social change. Putting babies born too soon high on the global, national and local health agenda requires consistent advocacy from all parties to build momentum and fuel progress.

**Fig. 7:**
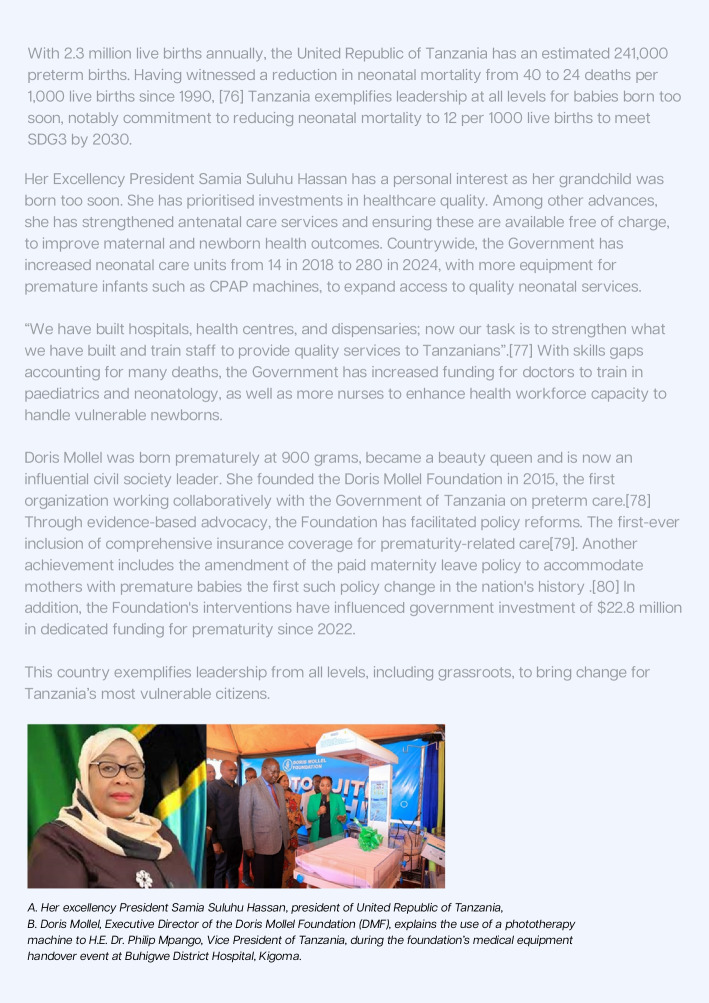
Country snapshot: Government-leadership at all levels in the United Republic of Tanzania for babies born too soon. References: [80–84]

Leadership is essential at all levels. Many countries have shown that strong leadership can achieve powerful results. Collective leadership around a shared vision can amplify commitments and accelerate transformative change. One such example of high-level political leadership is the Global Leaders Network for Women’s, Children’s, and Adolescents’ Health (GLN), [77] chaired by H.E. President Cyril Ramaphosa of South Africa and supported by the Partnership for Maternal, Newborn and Child Health (PMNCH) [78]. The initiative unites Heads of State and Government to drive faster progress for the most vulnerable, and to meet targets on maternal (SDG 3.1), neonatal (SDG 3.2.2), and adolescent health, particularly in sub-Saharan Africa. H.E. Samia Suluhu Hassan, President of Tanzania, is a member of the Global Leaders Network, exemplifying leadership for improving maternal and newborn health outcomes, including new financing initiatives (Fig. 7) [79].

Maternal, newborn, and child health must remain a political priority for the future we aspire to create. However, this issue is currently absent from the United Nations Pact for the Future, which aims to serve as a blueprint for the post-2030 development agenda. [85] Greater prioritisation of sexual, reproductive, maternal, newborn, child, and adolescent health is imperative. Immediate action and course correction are also needed to maximise the remaining years of the SDGs, and keep the issue healthy starts at the heart of the post-SDG framework

Investing in women’s health and caring for small and vulnerable newborns yields long-term benefits in human capital, accelerating national development, improving outcomes for families and future generations worldwide.

## Data Availability

All data is available in the paper or in supplementary files. Additional information is available at www.borntoosoonaction.org.
